# Multicenter prospective study of dedicated breast positron emission tomography (dbPET) for breast cancer: examination in preoperative patients

**DOI:** 10.1186/s12880-026-02307-1

**Published:** 2026-04-20

**Authors:** Youko Satou, Yuki Nakagami, Kazuyoshi Suga, Yamaguchi Breast Cancer Study Group (YBC)

**Affiliations:** 1 Department of Gastroenterological, Breast and Endocrine Surgery, School of Medicine, Yamaguchi University, 1-1-1 Minamikogushi, Ube-shi, Yamaguchi-ken, 755–8505 Japan; 2 Sasaki Medical Plaza, 9-13 Sentocyo, Yamaguchi-shi, Yamaguchi-ken, 753 − 0076 Japan; 3 Health Data Science Lab, Faculty of Data Science, Shimonoseki City University, 2-1-1 Daigaku-cho, Shimonoseki-shi, Yamaguchi-ken, 751–8510 Japan; 4 Department of Radiology, St. Hill Hospital, 3-7-18 Imamurakita, Ube-shi, Yamaguchi-ken, 755 − 8505 Japan

**Keywords:** Dedicated breast positron emission tomography (dbPET), Breast cancer, Standardized uptake value (SUVmax)

## Abstract

**Background:**

Dedicated breast positron emission tomography (dbPET) was developed to detect breast cancers smaller than those detectable using whole-body PET (wbPET). Although several studies have explored the use of dbPET, clear criteria for identifying which patients would benefit most from this modality are lacking. Our objective was to determine which patient groups would benefit most from dbPET and how it should be utilized. We conducted a multicenter, prospective exploratory study to investigate how the dbPET maximum standardized uptake values (SUVmax) correlate with patients’ clinical characteristics, other imaging modalities, and pathological findings of the lesions.

**Methods:**

In total, 219 patients with breast cancer (median age [range], 58.0 [30–83] years) were included in this study. The enrolled patients were divided into three groups (primary care, neoadjuvant therapy, postoperative follow-up patients). In this research, we examined the primary care group (*n* = 92). To investigate which patient groups benefit from dbPET, we examined which factors influence and correlate with dbPET SUVmax. Depending on the items being compared, correlation analysis, Wilcoxon signed rank test was used to examine the following items. Which factors (physical factors, pathological characteristics, etc.) correlate with dbPET SUVmax, differences between dbPET and other imaging examination (detection rate, etc.), and whether dbPET SUVmax L/H ratio (Lesion-to-Healthy (normal) site dbPET SUVmax ratio) incorporating dbPET SUVmax from healthy (normal) mammary gland tissue are necessary for evaluating dbPET SUVmax in lesion areas.

**Results:**

dbPET SUVmax in healthy(normal) mammary gland tissue were strongly associated with background mammary density observed on mammography (MMG) examination (positive correlation, *p*<0.05). Ki-67 showed the strongest positive correlation with both the lesion-site dbPET SUVmax (r=0.56, R^2^=0.31, *p*<0.05) and the dbPET SUVmax L/H ratio (lesion-to-healthy (normal) -site ratio) (r=0.47, R^2^=0.22, *p*<0.05). Additionally, the tissue grade, MMG and ultrasonography categories were positively correlated with dbPET SUVmax. Regarding the lesion detection rate, dbPET identified 100% of the lesions, including benign findings. The dbPET SUVmax L/H ratio showed a trend nearly identical to that of the dbPET SUVmax.

**Conclusions:**

dbPET demonstrated higher detection capabilities than other imaging tests and showed a strong correlation with tissue malignancy. Therefore, they were suggested to be potentially useful for distinguishing benign findings from malignant lesions that are difficult to differentiate using other imaging tests. The dbPET SUVmax of healthy mammary gland tissue were presumed to correlate with the amount of mammary gland tissue within the breast. However, no significant differences were observed in the correlations between the dbPET values and the L/H ratio and the individual parameters. In this study, it remained unclear whether the dbPET values of normal breast tissue should be taken into account when evaluating dbPET values.

**Supplementary Information:**

The online version contains supplementary material available at 10.1186/s12880-026-02307-1.

## Background

Breast cancer is the most common type of cancer worldwide. According to World Health Organization Global Breast Cancer Initiative data, the incidence and mortality rates of breast cancer are projected to increase by 38% and 68%, respectively, by 2050. Compared to the United States and Europe, the incidence rate of breast cancer is approximately half in Asia. However, mortality rates vary depending on the country and local conditions. Global medical countermeasures are required to address the increasing incidence of breast cancer and the disparity in mortality rates among countries and regions [1, 2]. Breast cancer is also the most prevalent cancer in Asia [3], including Japan. In Japan, it has been reported to have a low mortality rate and high morbidity rate [4]. Breast cancer treatment is constantly evolving, and treatment methods differ significantly depending on the characteristics of the cancer, stage, and histology. Dedicated breast positron emission tomography (dbPET) was specifically developed to detect small breast cancers. In Japan, this modality was covered by insurance from July 2013 onwards [5]. Numerous studies have assessed its usefulness and compared its breast cancer detection rates with those of other imaging modalities such as magnetic resonance imaging (MRI) and ultrasonography (US) [6–12]. These studies have also explored its ability to differentiate benign from malignant findings [13–16], assess the histological characteristics of breast cancer, and evaluate the effectiveness of neoadjuvant therapy [17–20].

However, clear criteria have not been established for identifying the patient groups for dbPET to be most useful; for example, there is no cut-off value for the dbPET maximum standardized uptake value (SUVmax) for distinguishing benign and malignant lesions. Although PET scanning is covered by insurance, it is unlike affordable and easily accessible imaging tests like MMG or US, this test is expensive even with insurance coverage and requires time-consuming preparation before the procedure.

Therefore, our objective was to determine which patient groups (primary care, neoadjuvant therapy, postoperative follow-up (screening for recurrence)) would benefit most from dbPET and how it should be utilized. To achieve this, we conducted a multicenter, prospective exploratory study to investigate how the dbPET SUVmax correlate with patients’ clinical characteristics, other imaging modalities, and pathological findings of the lesions.

## Methods

### Study design

This was a multicenter prospective observational study. Patients diagnosed with breast cancer at the time of the study and those who provided informed consent were enrolled.

The inclusion criteria were as follows: (1) age ≥ 20 years, (2) both male and female participants, and (3) patients who agreed to participate in this study of their own will and provided written consent. The exclusion criteria were (1) inability to maintain rest in the abdominal position and (2) pregnancy or suspected pregnancy at the time of dbPET inspection.

**Fig. 1:**
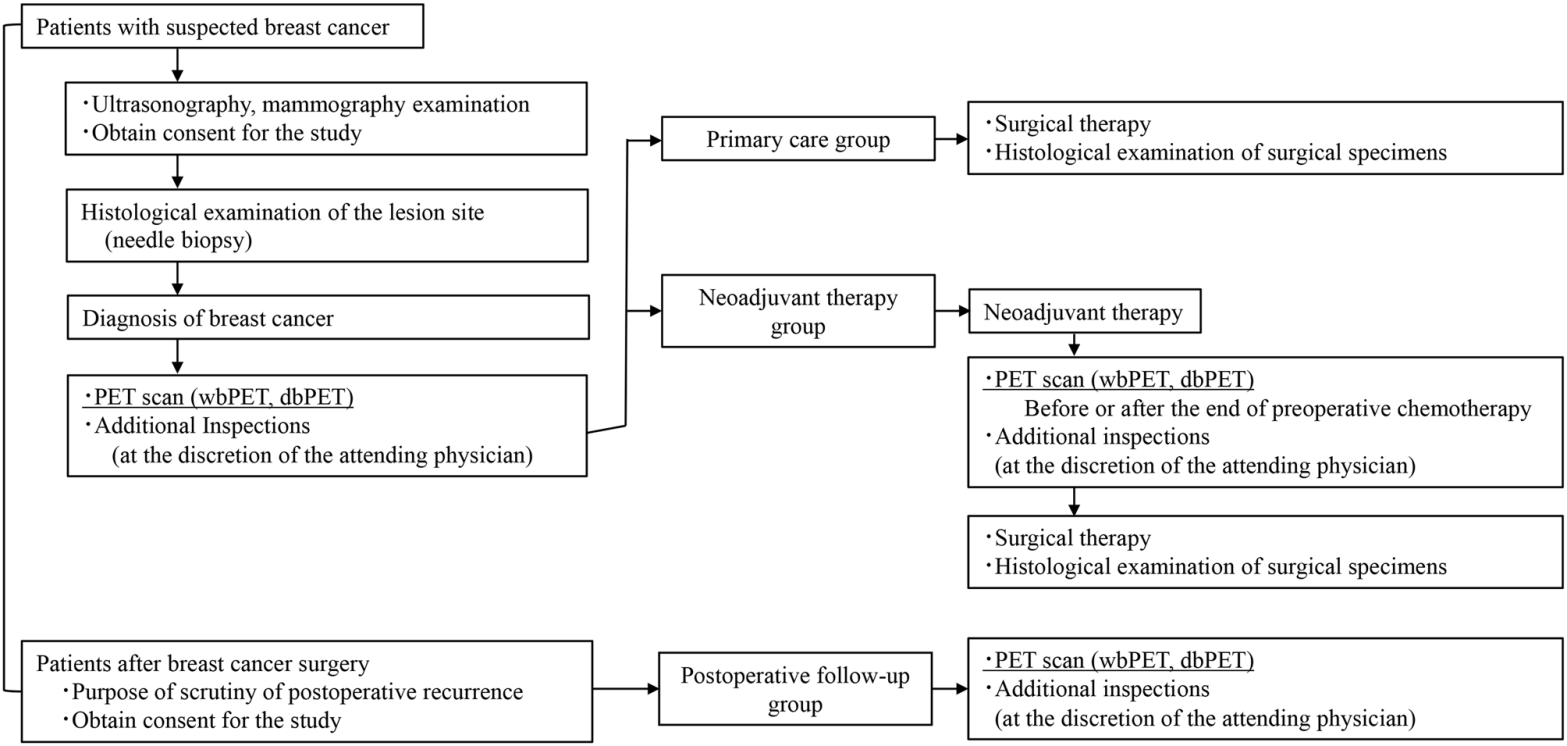
Study design

Overall, 219 patients with breast cancer who consented to participate in the study between March 1, 2020, and December 31, 2023, were enrolled. The patients were divided into three groups: preoperative, postoperative, and preoperative chemotherapy. This is because the purpose, timing, and accompanying imaging examinations for dbPET vary depending on the patient group. Mammography (MMG), US, whole-body PET (wbPET), and dbPET were performed preoperatively in all patients. Furthermore, contrast-enhanced computed tomography (CT) and MRI were performed at the discretion of the attending physician. (Fig. 1).

**Fig. 2:**
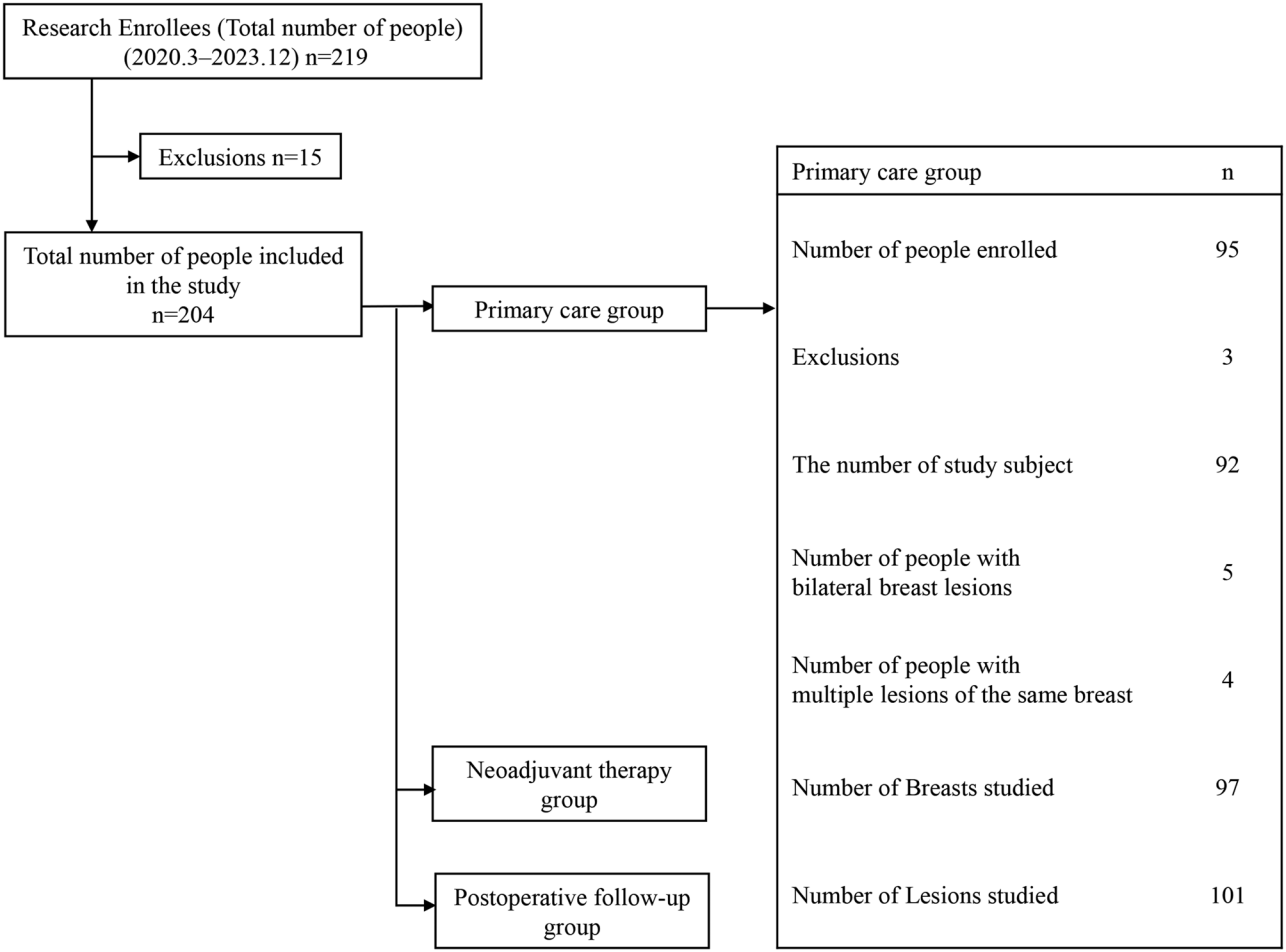
Registered patient allocation

This study focused on the initial treatment groups (*n *= 95). Statistical analysis and interpretation were difficult for neoadjuvant therapy group (*n* = 31) due to the small number of cases and for postoperative follow-up group (*n* = 78) due to the small number of cases with findings. Therefore, neoadjuvant therapy group and postoperative follow-up group were excluded from the scope of this study (Fig. 2).

### Diagnostic imaging methods

#### Ring-shaped dbPET scanner

All dbPET examinations were performed on the same day as the wbPET examinations and continued after the wbPET examinations were completed. Patients were instructed to fast for at least 4 h prior to receiving the fluorodeoxyglucose F18 (FDG) injection (dose, 3–3.7 MBq/kg). Subsequently, 1 h later, a wbPET was performed, followed immediately by a dbPET. We used a ring-shaped dbPET scanner (Elmammo, Shimadzu Corporation, Kyoto, Japan) with 36 detector modules (12 per ring) arranged in three continuous rings for each breast, with the patients in the prone position. The detector consisted of layers of a 32 × 32 cerium-doped lutetium gadolinium oxyorthosilicate crystal array (crystal size, 1.44 × 1.44 × 18 mm), light guide, and 64-channel position-sensitive photomultiplier tube. The field of view was 185 × 156.5 mm; with a scan time of 7 min per bed position. The acquired data were reconstructed into 236 × 236 matrix images (pixel size, 0.78 × 0.78 mm) using a three-dimensional dynamic row-action maximum likelihood algorithm. dbPET image evaluation and quantification of SUVmax were performed using the Xeleris workstation version 1.1452 (GE Healthcare, Little Chalfont, UK). Regions of interest were delineated within the primary tumor on attenuation-corrected FDG-PET images and within the ipsilateral normal breast tissue for background uptake, and the SUVmax was calculated. Attenuation correction for dbPET examinations was performed treating the breast tissue under examination as homogeneous soft tissue mixed with breast tissue and adipose tissue. The dbPET SUVmax L/H ratio (lesion-to-healthy (normal) mammary gland tissue ratio) was defined as the dbPET SUVmax of the lesion divided by that of the background (healthy(normal) mammary gland tissue) breast tissue.

#### wbPET/CT scanner

wbPET/CT was performed using the Biograph Horizon TrueV FDG-PET/CT system (Siemens Medical Solutions, Knoxville, TN, USA). The system had 52 detector rings consisting of 160 blocks. Each block contained an array of 13 × 13 lutetium oxyorthosilicate crystals (4 × 4 × 20 mm) covering an axial field of view (FOV) of 221 mm and a transaxial FOV of 690 mm. A CT scan was performed for attenuation correction (130 kV; 15–70 mA; tube rotation time, 0.6 s per rotation; pitch, 1; transaxial FOV, 700 mm; and section thickness, 5 mm).

### Imaging evaluation and diagnosis

In this study, image reading and evaluation were performed by breast specialists for US and MMG and by radiologists for CT, MRI, and PET examinations. Evaluation and diagnosis were performed according to the corresponding guidelines [22–24].

### Pathological diagnosis

In this study, histopathological diagnosis was established by a pathologist using needle biopsy specimens at the time of breast cancer diagnosis and surgical specimens based on the General Rules for Clinical and Pathological Recording of Breast Cancer (18th Edition, Japanese Breast Cancer Society) [21].

### Statistical analyses

Continuous and categorical variables were expressed as median values (interquartile ranges) and frequencies, respectively. For analyzing the correlation between two groups using continuous scales, regression lines and correlation coefficients were employed.

For analyzing the comparison between two or more groups, the Wilcoxon test with the Bonferroni correction was used. Statistical tests were two-sided, with significance set at *p* < 0.05. Statistical analyses were performed using JMP Pro 16 (SAS Institute, Cary, NC, USA).

## Results

Patient and tumor characteristics of the primary car group is shown in Tables 1 and 2.

**Table 1:** Patient and tumor characteristics of the primary care group

	*n*		*n*
Number of Enrolled Patients	96	Pathology	
Number of Excluded Patients	4	Benign	3
		Fibroadenoma	2
Number of Studied Patients	92	Mastopathy	1
Number of Patients with Bilateral breast lesions	5	DCIS	6
Number of Patients with Multiple lesions of the same breast	4	LCIS	0
Number of breasts studied	97	Microinvasive carcinoma	1
Number of lesions studied	101	IDC	69
		Tubule-forming type	16
Age (years) (median(range))	57.5 (30–83)	Solid type	6
BMI (kg/m^2^) (median(range))	21.5 (13.5–35.3)	Scirrhous type	46
Menopause status		N/A	1
Pre-menopause	36	Special	22
Post-menopause	56	Invasive Lobular carcinoma	10
		Mucinous carcinoma	7
cT		Apocrine carcinoma	3
cT1a	0	Invasive micropapillary carcinoma	2
cT1b	15		
cT1c	39	Subtypes	
cT2	44	ER+/PgR+/HER2-	86
cT3	1	ER+/PgR+/HER2+	5
N/A	2	ER-/PgR-/HER2+	2
		ER-/PgR-/HER2-	5
pT (mm) (median [interquartile rage])	18 (3–70)		
pT		Ki-67 (%)	
Benign	3	< 20	41
Tis	6	20–50	37
Tmi	1	> 50	12
T1a	2	N/A	11
T1b	11		
T1c	40		
T2	35		
T3	3		

BMI, body mass index; DCIS, ductal carcinoma in situ; IDC, invasive ductal carcinoma; LCIS, lobular carcinoma in situ; ER, estrogen receptor; PgR, progesterone receptor; HER2, human epidermal growth factor receptor type 2

**Table 2:** dbPET SUVmax value of healthy mammary tissue

Primary care group	*n*	median (range)	Healthy mammary gland dbPET SUVmax (median [range])
Number of people with studied patient number	92		
Bilateral lesion number	5		
Multiple lesion number	7		
Studied Breast number	101		
Age	92	57.5 (30.0–83.0)	
BMI	92	21.5 (13.5–35.3)	
Menopause Status	92		
Premenopausal	36		1.6 (1.0–2.8)
Postmenopausal	56		1.3 (0.9–2.8)
MMG Breast density			
Fatty	10		1.1 (0.9–1.3)
Scattered	49		1.4 (0.9–2.4)
Heterogeneous Dense	29		1.6 (1.2–2.8)
Extremely Dense	5		1.6 (1.5–2.1)
N/A	8		

BMI, body mass index; MMG, mammography

In a dbPET, the healthy (normal) mammary gland tissue outside the lesion site exhibits a baseline dbPET SUVmax, representative of its background, and normal metabolic activity. To examine whether background (healthy(normal) mammary gland) dbPET SUVmax should be considered when evaluating lesion dbPET SUVmax, we examined how background dbPET SUVmax correlate with individual patient differences (age, body mass index [BMI], menopause status, and MMG background breast density).

**Fig. 3:**
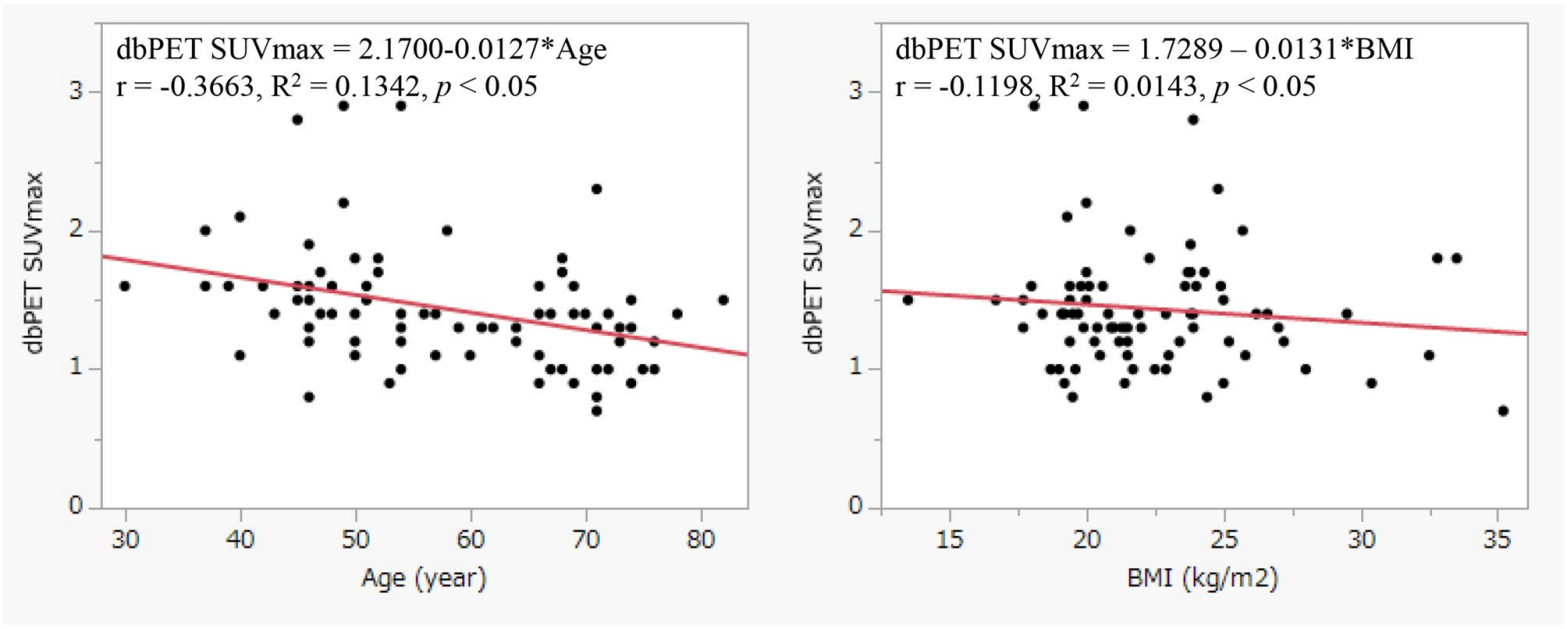
Correlation between healthy mammary site dbPET SUV max and physical information (Age, BMI)

**Fig. 4:**
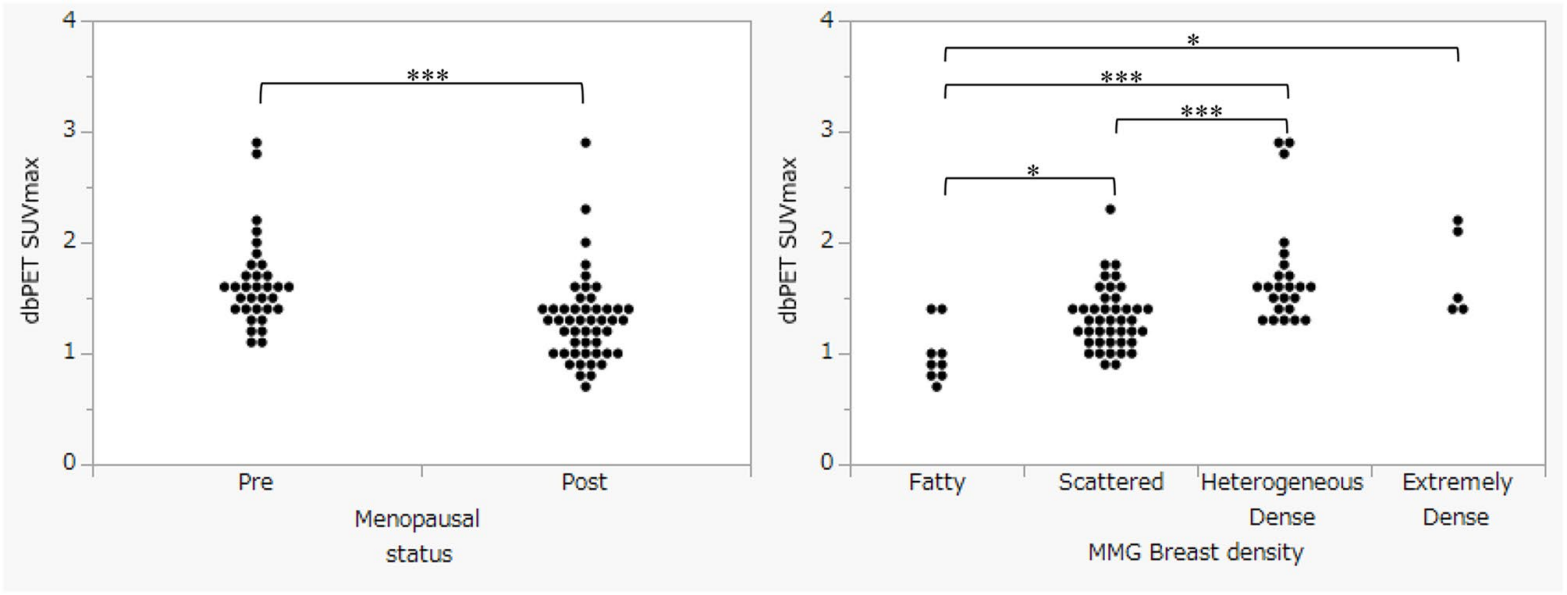
Comparison between healthy mammary site dbPET SUV max, physical information (menopausal status) and MMG breast density

The dbPET SUVmax of the healthy (normal) mammary sites are shown in Table 2; Figs. 3 and 4. The healthy (normal) mammary gland site dbPET SUVmax (background dbPET SUVmax) were negatively correlated with age (*p* < 0.05, R^2^ = 0.1342) and menopausal status (*p* < 0.001). And positively correlated with MMG breast density. BMI and dbPET SUVmax were less closely related. (*p* < 0.05, R^2^ = 0.0143)

**Fig. 5:**
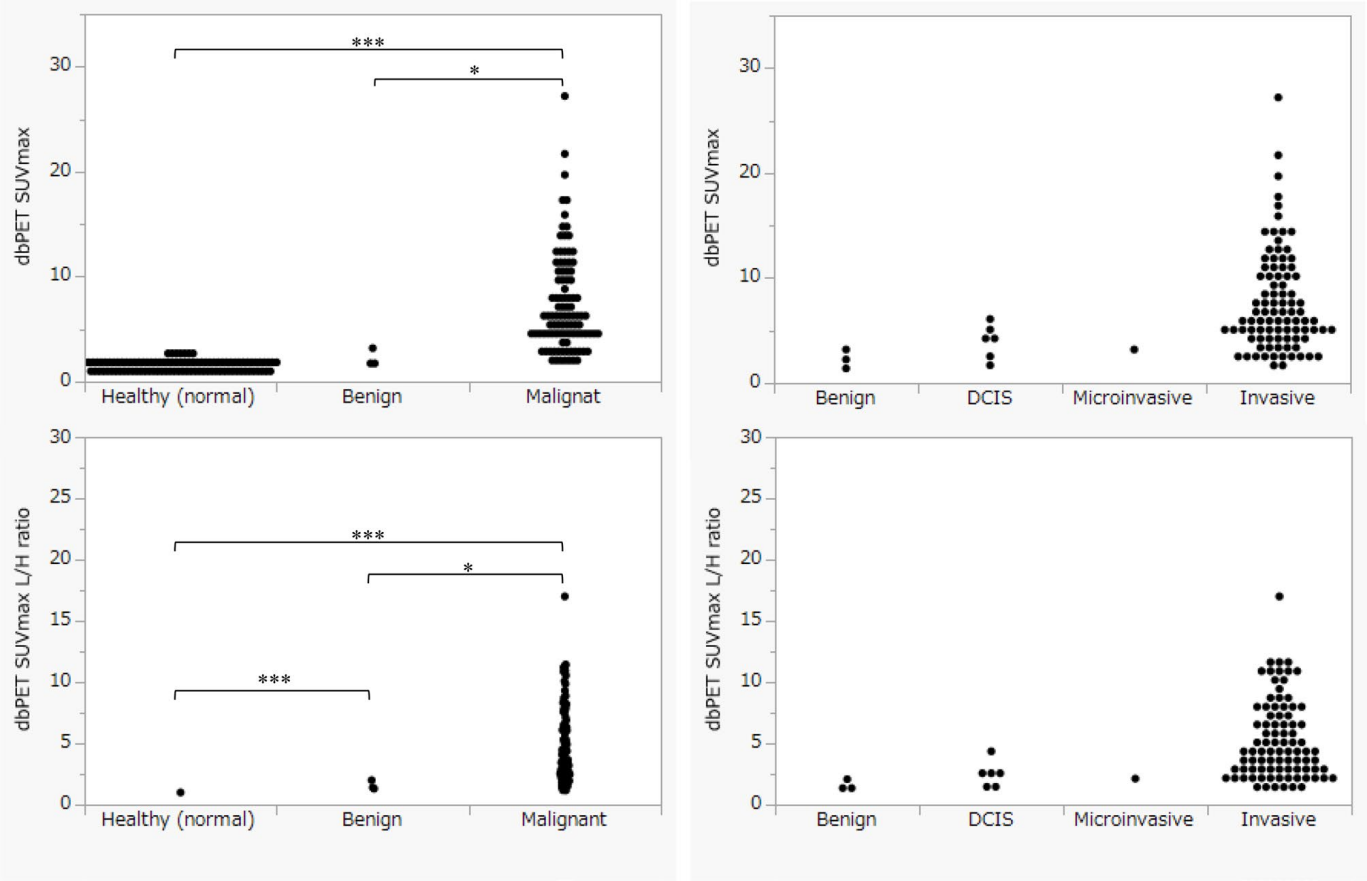
Comparison between dbPET SUV max, healthy(normal) mammary glands, benign findings, and malignant lesions

Figure 5 shows the differences in the dbPET SUVmax and dbPET SUVmax L/H ratio (lesion-to-healthy (normal) -site ratio) for healthy (normal) mammary gland sites, benign findings, malignant lesions, and subdivisions of malignant lesions. Benign findings and malignant lesions were diagnosed based on histopathological findings of biopsy or surgical specimens. The results in suggest a tendency for dbPET SUVmax and dbPET SUVmax L/H ratio, to differ between benign findings and malignant lesions (such as DCIS and microinvasive cancer).

**Fig. 6:**
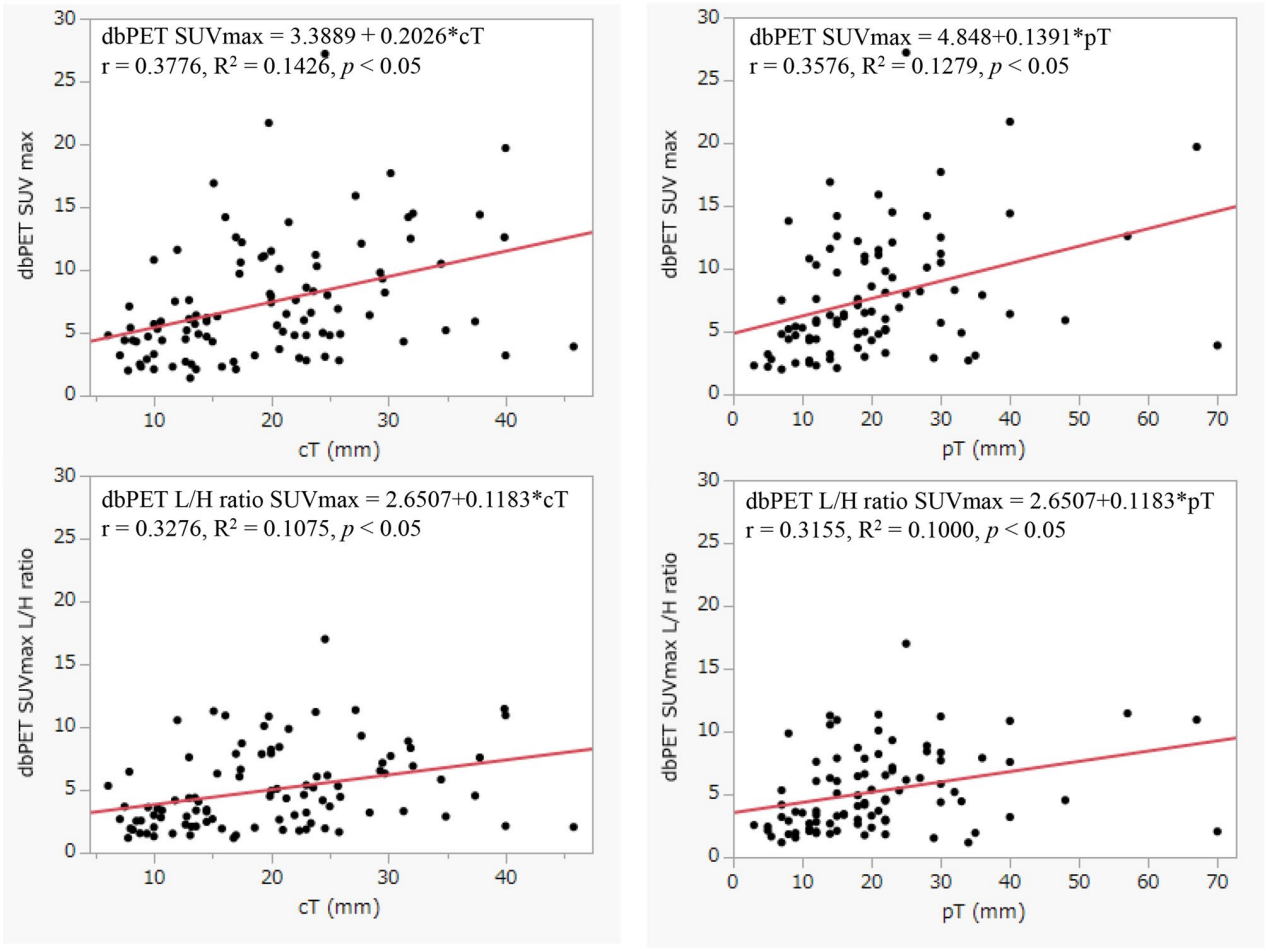
Correlation between dbPET SUVmax, dbPET SUVmax L/H ratio, cT (mm) and pT (mm)

**Fig. 7:**
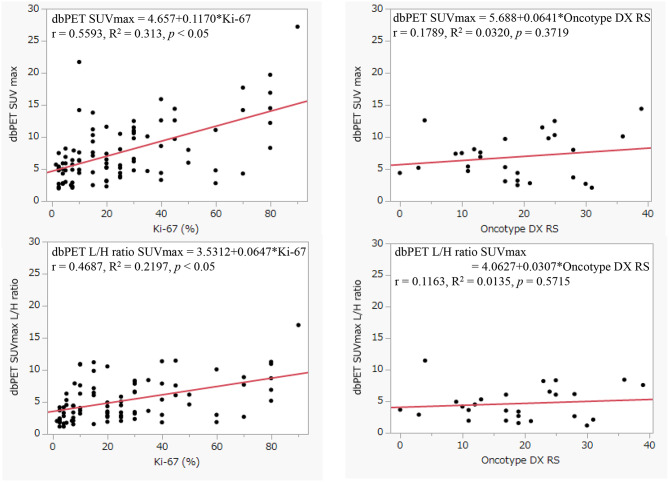
Correlation between dbPET SUVmax, dbPET SUVmax L/H ratio, Ki-67(%) and Oncotype DX RS

Figures 6 and 7 shows the relationship among cT (mm), pT (mm), Ki-67 (%), and Oncotype DX RS. Ki-67 (%) values were shown to correlate more strongly with dbPET SUVmax (R^2^ = 0.313) than tumor size. (cT: R^2^ = 0.1426, pT: R^2^ = 0.1279) No clear relationship was observed with the Oncotype DX Recurrence Score with dbPET SUVmax. (R^2^ = 0.0320) The dbPET SUVmax L/H ratio showed a similar trend.

**Fig. 8:**
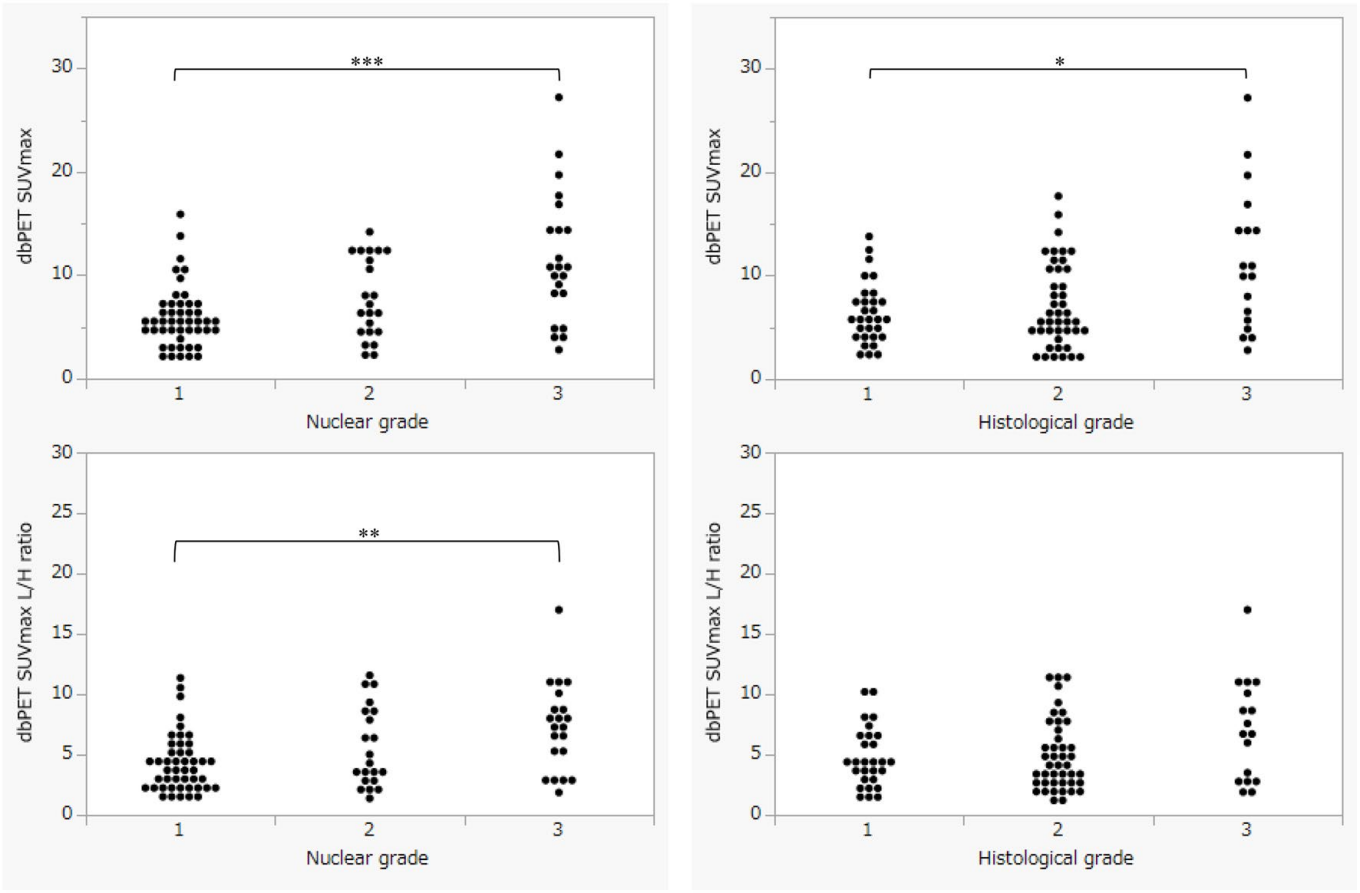
Comparison between dbPET SUVmax, dbPET SUVmax L/H ratio, nuclear grade and histological grade

**Fig. 9:**
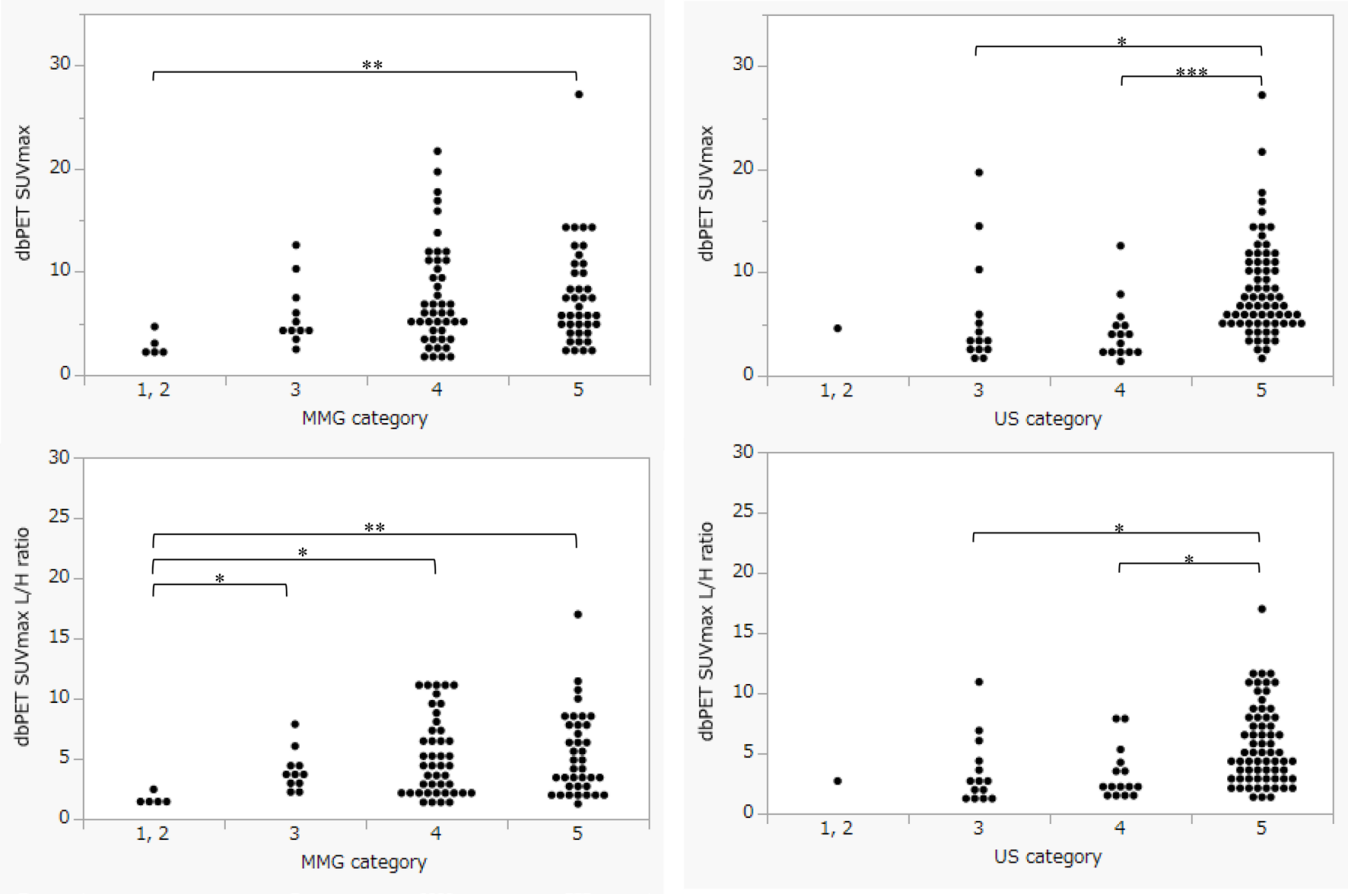
Comparison between dbPET SUVmax, dbPET SUVmax L/H ratio, MMG category, and US category

The relationship between nuclear grade, histological grade, MMG category, and US category is shown in Figs. 8 and 9. All items were positively correlated with increased grades and malignant findings. The dbPET SUVmax L/H ratio showed a similar trend. However, regarding the correlation between MMG categories and dbPET SUVmax, (Fig. 9) the use of the dbPET SUVmax L/H ratio made the differences in dbPET values between categories more pronounced.

Table 3 shows the lesion detection rates for each imaging modality (MMG, US, contrast-enhanced CT, contrast-enhanced MRI, wbPET, and dbPET), with a detection rate of 100% for contrast-enhanced MRI and dbPET.

**Table 3:** Lesion detection rate for each imaging test

		MMG	US	CT	MRI	wbPET	dbPET
Number of inspections performed	(*n*)	101	100	88	13	101	101
	(%)	100.0	99.0	93.2	12.9	100.0	100.0
Number of detected lesions	(%)	95.1	99.0	93.2	100.0	96.0	100.0
Number of non-detected leisons	(n)	5	1	6	0	4	0
	(%)	4.9	1.0	6.8	0.0	4.0	0.0
Pathology of non-detected lesions (n)	Benign	1		2		2	
	DCIS	2	1	1		2	
	IDC	2		2			
	Special			1			

MMG, mammography examination; US, ultrasound examination; CT, enhanced computed tomography; MRI, enhanced magnetic resonance imaging examination; wbPET, whole-body positron emission tomography; dbPET, dedicated breast positron emission tomography; DCIS, ductal carcinoma in situ; IDC, invasive ductal carcinoma; Special, special type invasive carcinoma

Table 4 summarizes the lesions that were not detected by each imaging test. dbPET successfully detected all lesions. All benign findings (*n* = 3) were detected using dbPET.

**Table 4:** List of non-detected lesions by an imaging test

Pathology		cT	mm	dbPET SUVmax	MMG (background breast density)	US	CT	MRI	wbEPT	dbPET
Benign	Mastopathy	T1b	10.0	2.1	ND (Heterogeneous)		ND	N/A	ND	
	Fibroadenoma	T1c	13.1	1.4			ND	N/A	ND	
	Fibroadenoma	T1c	18.6	3.2			N/A	N/A		
Malignant	Subtype									
DCIS	Triple negative	T1c	11.6	2.3			ND		ND	
DCIS	Luminal	N/A		4.6		ND			ND	
DCIS	Luminal	T1c	14.5	4.7	ND (Heterogeneous)			N/A		
DCIS	Triple negative	T1c	17.0	2.1	ND (N/A)					
Special (Apocrine)	Luminal	T1b	8.8	2.5	ND (Extremely)					
IDC (Scirrhous)	Luminal	T1b	8.9	2.2			ND			
Special (Lobular)	Luminal	T1b	9.4	2.9	ND (Heterogeneous)		N/A	N/A		
IDC (Scirrhous)	Luminal	T2	23.0	8.6			ND	N/A		
Special (Mucinous)	Luminal	T2	37.4	5.9			ND	N/A		

DCIS, in situ ductal carcinoma; Special, special type; IDC, invasive ductal carcinoma; cT, clinical tumor size; dbPET, dedicated breast positron emission tomography; MMG, mammography; US, ultrasonography; CT, enhanced computed tomography; MRI, magnetic resonance imaging; wbPET, whole-body positron emission tomography; dbPET, dedicated breast positoron emission tomography; Heterogeneous, Heterogeneous dense, Extremely, Extremely dense

ND, non-detected lesion; N/A, not inspected

## Discussion

The objective of our study on dbPET imaging is to determine which patient groups would benefit most from dbPET imaging and how it should be used to best leverage its unique characteristics. To this end, in this study, we conducted an exploratory study to identify the factors that correlate with and influence dbPET SUVmax values.

In relation to previously reported studies and our current study, dbPET SUVmax in healthy (normal) mammary glands (background SUVmax) were negatively associated with age and menopausal status and positively associated with MMG background mammary density, which is consistent with the findings of Shimizu et al. [25]. Based on these results, it is inferred that dbPET SUVmax in healthy (normal) mammary gland tissue (background dbPET SUVmax) reflect the amount of mammary glands tissue within the breast. Therefore, it is speculated that differences in dbPET SUVmax within healthy (normal) mammary gland tissue may influence the detection of lesions and the measured dbPET SUVmax of lesions.

Some reports indicate that incorporating or combining background dbPET SUVmax (healthy(normal) mammary gland tissue dbPET SUV max) can improve the accuracy of distinguishing between benign and malignant lesions detected by dbPET [13]. 

Both MMG and US are performed in clinical practice. Many studies have reported MMG and US lesion detection rates > 90% [7, 13–16], our research yielded similar results too. (Tables 3 and 4). If the lesion detection rate exceeds 90%, it is reasonable to assume that MMG and US, when used for screening, are capable of detecting nearly all lesions.

Many studies have reported the differentiation of benign from malignant lesions using dbPET, focusing on morphological features, integration with other imaging modalities, and cutoff values detected using ROC curves [11–16], and the ROC curve is more sensitive when the background dbPET SUVmax (healthy(normal) site mammary gland tissue dbPET SUVmax) is considered in dbPET examinations [13]; however, no report has explicitly stated the cutoff value. Recently, there have been reports of using dbPET to differentiate between benign and malignant lesions using deep learning and artificial intelligence technology [26, 27]. There are also numerous reports on neoadjuvant chemotherapy cases regarding the assessment of chemotherapy efficacy and the determination of pCR (pathological complete response). However, due to increased and fluctuating dbPET SUVmax caused by treatment-induced tissue inflammation and heterogeneity in breast cancer tissue, the usefulness of dbPET imaging remains a subject of debate [19, 20, 28]. 

In this study, it was difficult to determine whether the dbPET SUVmax of healthy (normal, background) mammary gland tissue should be taken into account.

However, although the dbPET SUVmax correlated with the proportion of healthy (normal) mammary gland tissue in the same way as the MMG, it demonstrated higher detection sensitivity for lesions than MMG. It was inferred that, unlike MMG, tissue malignancy plays a greater role in detecting lesions in dbPET than tissue density or tumor size. A previous study reported that dbPET SUVmax were most positively associated with Ki-67 (%) [7]. Our research yielded similar results. (Fig. 7)

This suggests that, unlike other imaging modalities where it is difficult to distinguish between benign findings and malignant small lesions, dbPET shows a strong correlation with histological grade rather than tumor size; therefore, dbPET may be useful in distinguishing between benign and malignant small lesions.

The utility of dbPET can be demonstrated in patients with dense breasts—where MMG has a lower lesion detection rate—as well as in patients with post-surgical breast deformities following partial mastectomy, where detecting lesions is as difficult with US as it is with MMG.　Furthermore, if dbPET prove to be a useful tool for distinguishing between benign and malignant lesions, there is potential for them to demonstrate value in detecting postoperative recurrence in cases where assessment via MMG or US is difficult—such as in breasts with postoperative scarring. In this context, if dbPET can be used to evaluate the distinction between tissue inflammation caused by chemotherapy and residual malignant lesions, as well as differences in their characteristics, this could potentially contribute to the assessment of pCR (pathological complete response) in neoadjuvant chemotherapy cases.

In this paper, we have not reported on cases of neoadjuvant chemotherapy group or the postoperative follow-up patient group because the analysis of these data was insufficient.

However, there are no published studies—such as our own—on dbPET imaging that are prospective, multicenter studies examining the utility of dbPET for detecting postoperative recurrence in breasts with postoperative deformities or scarring, where lesions are difficult to detect using MMG or US.

Limitations of this study, first, the small sample size and potential bias make objective analysis difficult. A definitive histopathological diagnosis is required for the tissue samples from the cases under study. It is particularly difficult to study benign findings. In this study, the collection of benign findings was largely limited to cases in which such lesions were identified incidentally. This is because, in Japan, dbPET scans are covered by insurance only for cases where breast cancer has already been diagnosed. For findings that are benign or suspected to be benign findings on MMG or US scans, the patient is placed under observation, and further examination such as dbPET or a biopsy is not performed. Therefore, we believe it will be necessary to explore new approaches in the future, such as expanding the scope of facilities included in the study (e.g., facilities that perform dbPET scans on a self-pay basis for health screening patients).

Furthermore, if we are to conduct a prospective evaluation of the utility of postoperative screening for recurrence, a long study period is necessary to ensure a sufficient number of subjects with significant findings (such as recurrent cases) for statistical analysis. This requires a large number of study participants and their cooperation, and results will not be available in the short term.

Regarding comparisons with other imaging tests, as a practical matter, decisions regarding additional imaging tests (such as CT and MRI scans) are left to the attending physician’s discretion. However, in order to obtain reliable case data for research purposes, we believe it is necessary to consider narrowing down the scope of imaging tests used for comparison with dbPET scans, or standardizing them through collaboration with research institutions.

We believe it will be necessary to make improvements and adjustments, including to the research design, going forward. The primary care group included in this study consists of patients for whom a definitive histological diagnosis of the lesion could be obtained. It is necessary to continue accumulating long-term data not only for the primary care group but also for other patient groups.

## Conclusion

dbPET examination has a higher detection rate compared to other imaging modalities. And dbPET SUVmax in healthy (normal) breast tissue increased proportionally to the amount of normal mammary gland tissue in breast. However, no clear difference in dbPET SUVmax was observed when evaluating lesion dbPET SUVmax with or without considering healthy (normal) mammary gland tissue dbPET SUVmax. dbPET SUVmax correlated most strongly with tissue malignancy (Ki-67) than tumor size, suggesting potential utility in cases where benign/malignant differentiation is difficult with other imaging tests.

## Supplementary Information

Below is the link to the electronic supplementary material.

Supplementary Material 1

## Data Availability

The data analyzed in the current study are available from the corresponding author upon reasonable request.

## Declarations

### ^5^Yamaguchi Breast Cancer Study Group (YBC)

YBC Group: Representative, Address, and Contact Information.

YBC Group Representative: Hiroaki Nagano^1,5^,

YBC Group Adress, Contact Information: ^1^Department of Gastroenterological, Breast and Endocrine Surgery, School of Medicine, Yamaguchi University, 1-1-1 Minamikogushi, Ube-shi, Yamaguchi-ken 755–8505, Japan.

Tel: +81-836-22-2264.

E-mail: ges_res@yamaguchi-u.ac.jp,

Mari Nabeya^1,5^, Lisa Suyama^1,5^, Yuka Yano^5,6^, Gaku Fukamitsu^5,7^, Takashi Arata^5,8^, Yoshinari Maeda^5,9^, Michinori Iwamura^5,10^, Satoshi Onitsuka^5,11^, Noriko Maeda^1,5^, Yukiko Nagashima^5,12^, Masakazu Fujii^5,13^, Nobuki Matsunami^5,14^, Shigeru Yamamoto^5,15^, Kimikazu Hamano^5,16^, Hiroaki Nagano^1,5^,

^1^Department of Gastroenterological, Breast and Endocrine Surgery, School of Medicine, Yamaguchi University, 1-1-1 Minamikogushi, Ube-shi, Yamaguchi-ken 755–8505, Japan.

^6^Department of Surgery, Saiseikai Shimonoseki General Hospital, 8-5-1, Yasuokacyo, Shimonoseki-shi, Yamaguchi-ken 759–6603, Japan.

^7^Fukamitsu Breast Clinic, 3-4-24, Kotobukicyo, Ube-shi, Yamaguchi-ken 755 − 0032, Japan.

^8^Department of Surgery, National Hospital Organization Iwakuni Clinical Center, 1-1-1, atagocyo, Iwakuni-shi, Yamaguchi-ken 740 − 0037, Japan.

^9^Department of Surgery, Shunan City Shinnanyo Hospital, 2-3-15, Mianomae, Shunan-shi, Yamaguchi-ken 746 − 0017, Japan.

^10^Department of Surgery, Yamaguchi Rosai Hospital, 1315-4, Ooazaonoda, Sanyoonoda-shi, Yamaguchi-ken, 756 − 0095, Japan.

^11^Department of Surgery, Yamaguchi Red Cross Hospital, 53-  1, Yahatababa, Yamaguchi-shi, Yamaguchi-ken 753 − 0092, Japan.

^12^Department of Breast Surgery, NHO Kanmon Medical Center, 1-1, Cyofusotoura-cyo, Shimonoseki-shi, Yamaguchi-ken 752–8510, Japan.

^13^Department of Breast Surgery, Yamaguchi Prefectural Grand Medical Center, 10077, Ooazaoosaki, Houfu-shi, Yamaguchi-ken 747–8511, Japan.

^14^Department of Breast Surgery, Shuto General Hospital, 1000-1, Kogaisaku, Yanai-shi, Yamaguchi-ken 742 − 0032, Japan.

^15^Department of Breast Surgery, JCHO Tokuyama Central Hospital, 1- 1, Kouda-cho, Syuunann-shi, Yamaguchi-ken 745–8522, Japan.

^16^Department of Surgery and Clinical Science, School of Medicine, Yamaguchi University, 1-1-1, Minamikogushi, Ube-shi, Yamaguchi-ken 755–8505, Japan.

## Abbreviations

dbPET: Dedicated breast positron emission tomography
wbPET: Whole-body positron emission tomography
SUVmax: Maximum standardized uptake value
MMG: Mammography
US: Ultrasonography
CT: Computed tomography
MRI: Magnetic resonance imaging
LBR: Lesion-to-background ratio
L/H ratio: Lesion site dbPET SUVmax /Healthy (normal) mammary gland tissue (background) dbPET SUVmax ratio
cT: Clinical tumor size (measured by ultrasonography, mm)
pT: Pathological tumor size (mm)
DCIS: Ductal carcinoma in situ
Oncotype DX RS: Oncotype DX recurrence score
pCR: Pathological complete response
