# Supplementary material for: Multicenter prospective study of dedicated breast positron emission tomography (dbPET) for breast cancer: examination in preoperative patients
Source: BMC Med Imaging. 2026 Apr 20;26:199. doi: 10.1186/s12880-026-02307-1 (PMC13094001; doi:10.1186/s12880-026-02307-1)
Supplement: Supplementary file 1 — Supplementary Material 1 [file 12880_2026_2307_MOESM1_ESM.pptx]

## Slide 1
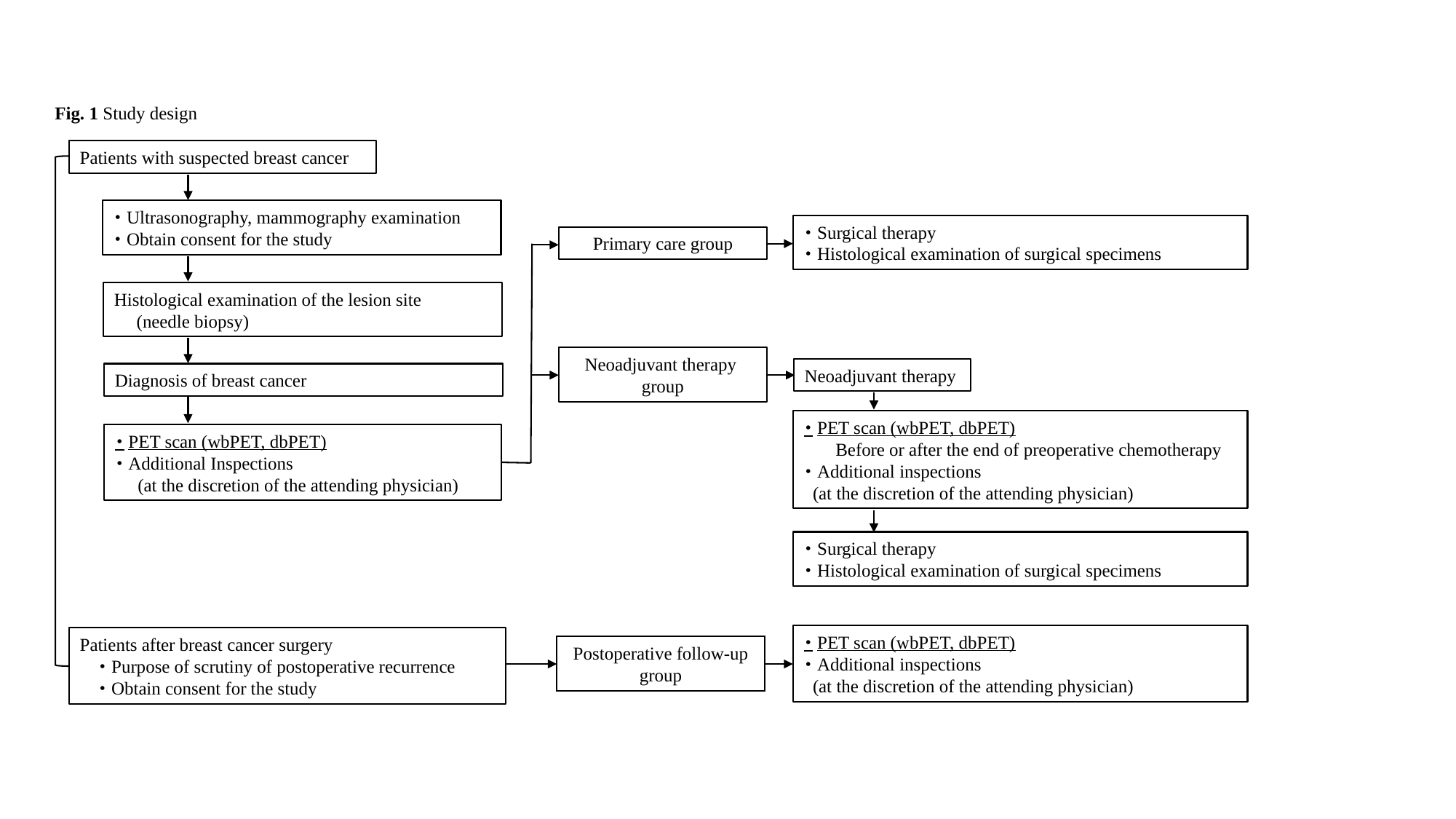

Fig. 1 Study design
Patients with suspected breast cancer
･Ultrasonography, mammography examination
･Obtain consent for the study
Histological examination of the lesion site
　(needle biopsy)
Diagnosis of breast cancer
･PET scan (wbPET, dbPET)
･Additional Inspections
 (at the discretion of the attending physician)
･Surgical therapy
･Histological examination of surgical specimens
Primary care group
Neoadjuvant therapy group
･PET scan (wbPET, dbPET)
 Before or after the end of preoperative chemotherapy
･Additional inspections
 (at the discretion of the attending physician)
･Surgical therapy
･Histological examination of surgical specimens
･PET scan (wbPET, dbPET)
･Additional inspections
 (at the discretion of the attending physician)
Patients after breast cancer surgery
　･Purpose of scrutiny of postoperative recurrence
　･Obtain consent for the study
Postoperative follow-up group
Neoadjuvant therapy

## Slide 2
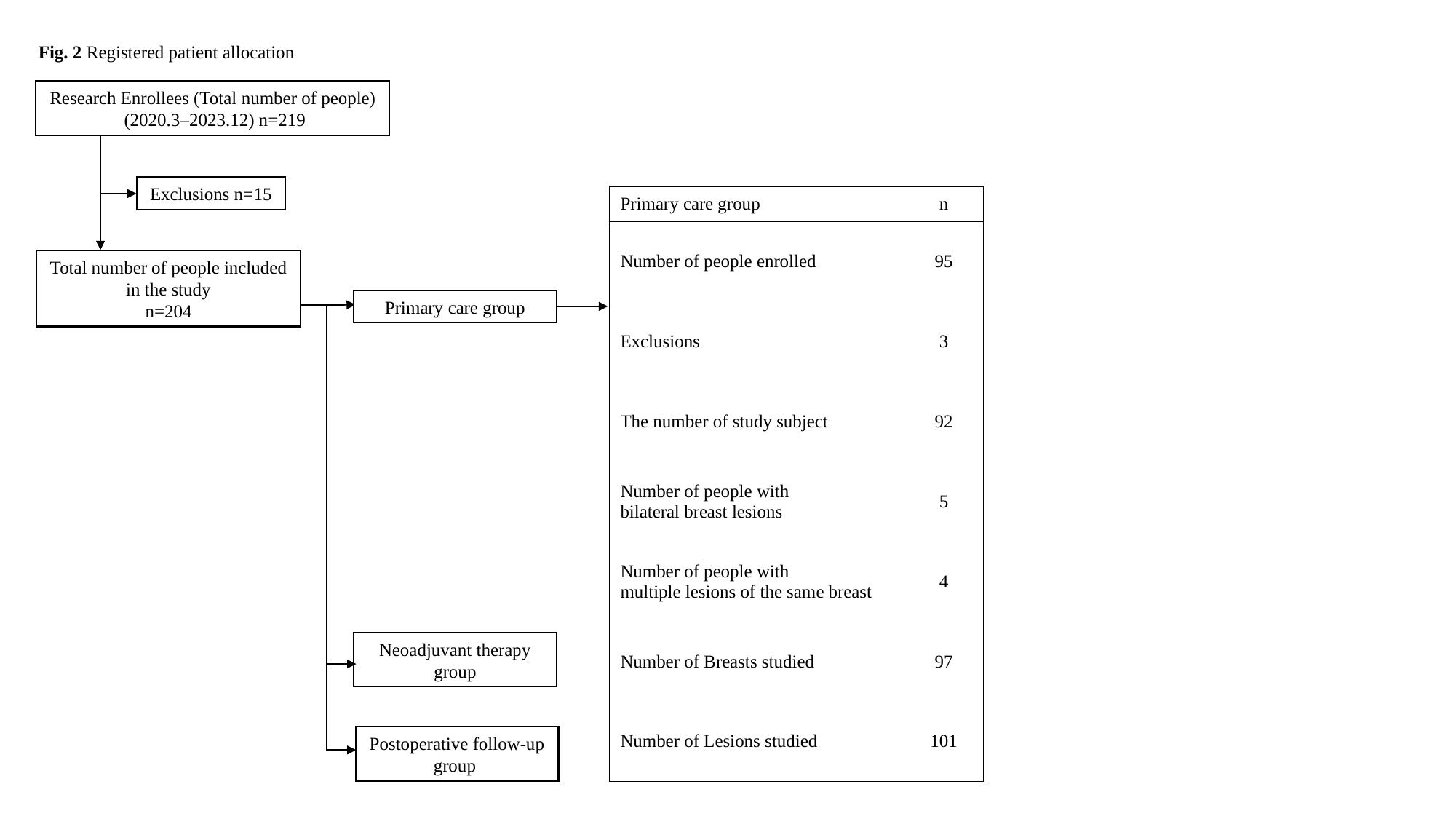

Fig. 2 Registered patient allocation
Research Enrollees (Total number of people)
 (2020.3–2023.12) n=219
Exclusions n=15
Total number of people included in the study
n=204
| Primary care group | n |
| --- | --- |
| Number of people enrolled | 95 |
| Exclusions | 3 |
| The number of study subject | 92 |
| Number of people with bilateral breast lesions | 5 |
| Number of people with multiple lesions of the same breast | 4 |
| Number of Breasts studied | 97 |
| Number of Lesions studied | 101 |
Primary care group
Neoadjuvant therapy
group
Postoperative follow-up group

## Slide 3
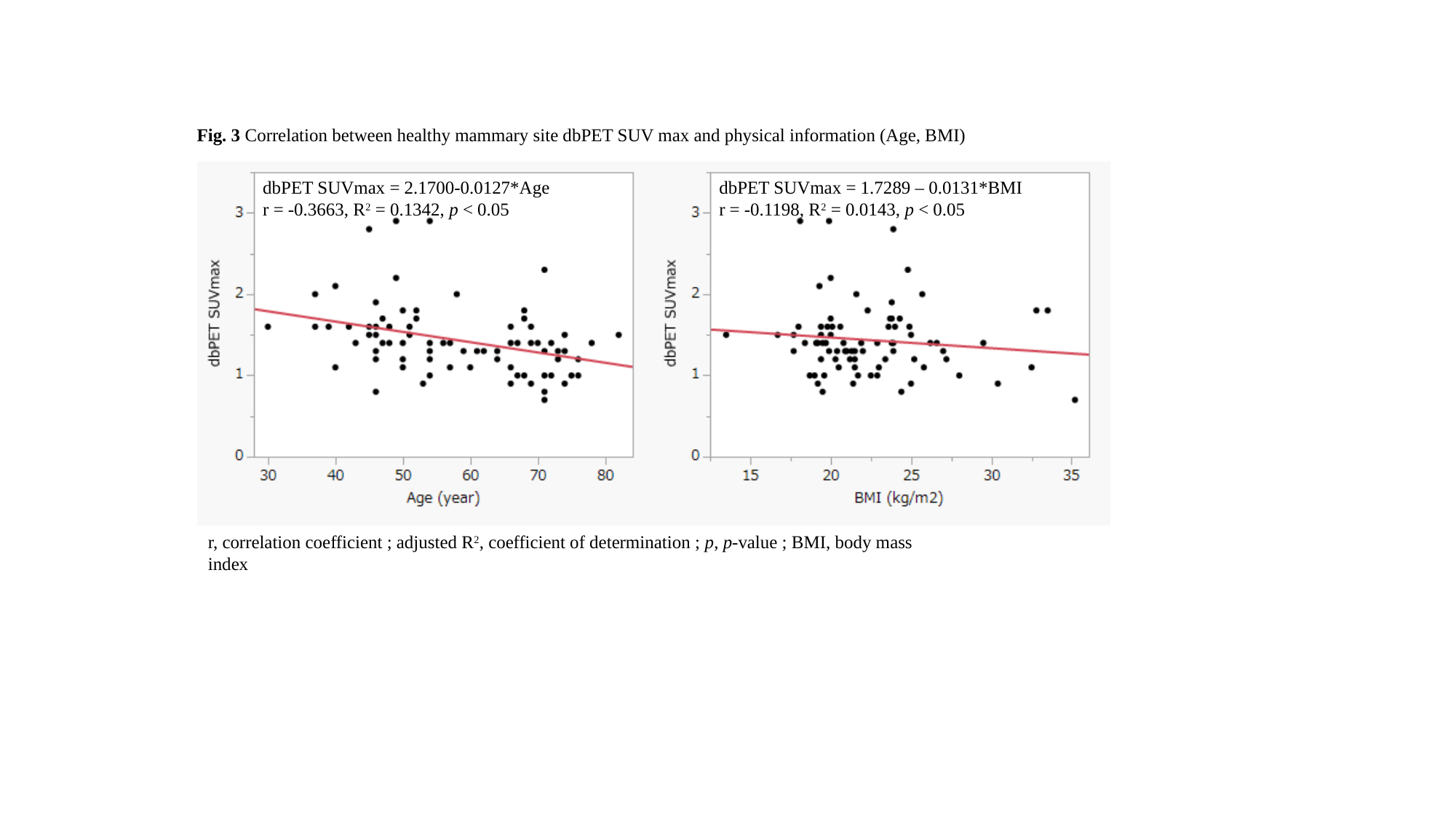

Fig. 3 Correlation between healthy mammary site dbPET SUV max and physical information (Age, BMI)
dbPET SUVmax = 1.7289 – 0.0131*BMI
r = -0.1198, R2 = 0.0143, p < 0.05
dbPET SUVmax = 2.1700-0.0127*Age
r = -0.3663, R2 = 0.1342, p < 0.05
r, correlation coefficient ; adjusted R2, coefficient of determination ; p, p-value ; BMI, body mass index

## Slide 4
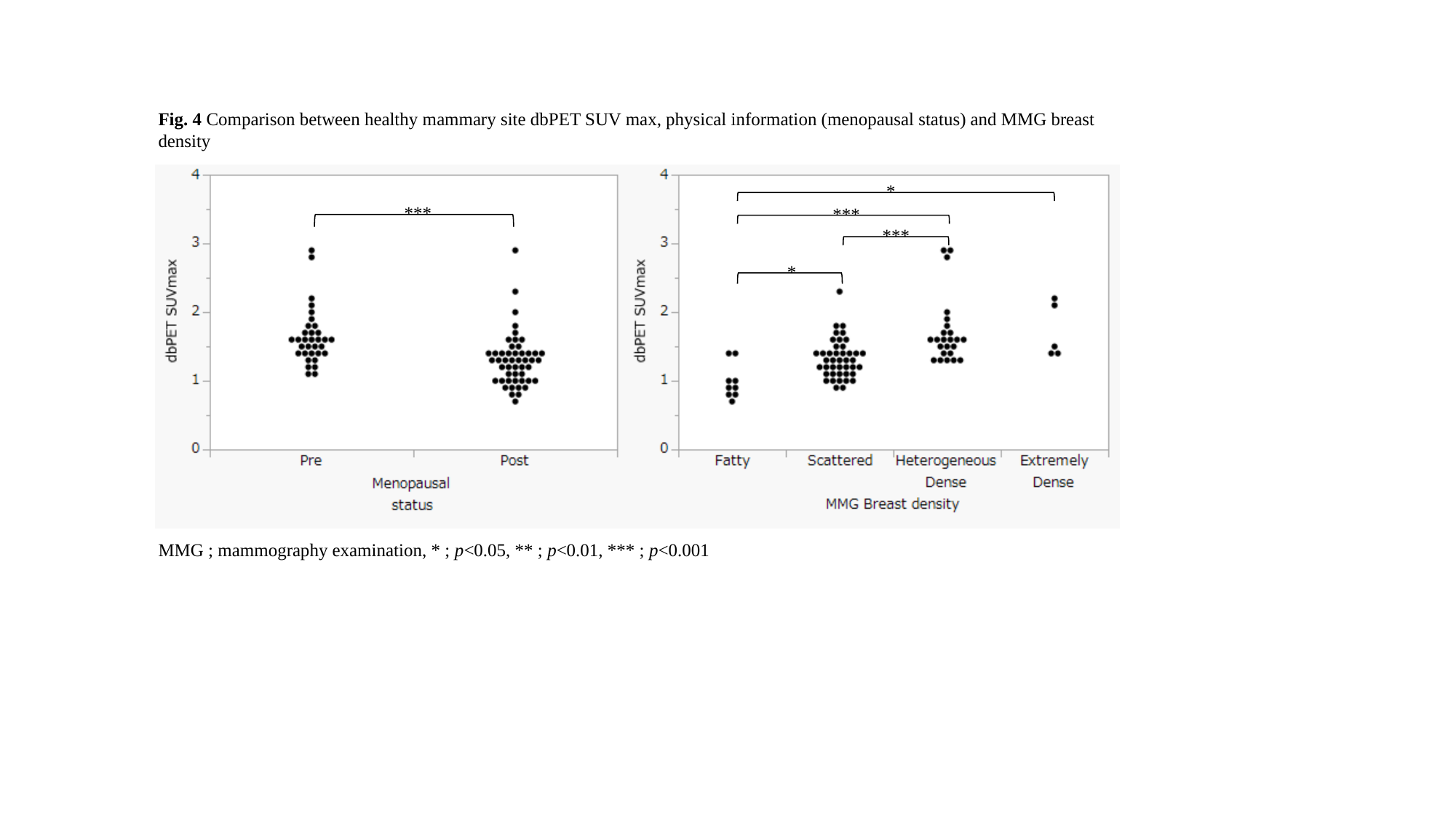

Fig. 4 Comparison between healthy mammary site dbPET SUV max, physical information (menopausal status) and MMG breast density
*
***
***
***
*
MMG ; mammography examination, * ; p<0.05, ** ; p<0.01, *** ; p<0.001

## Slide 5
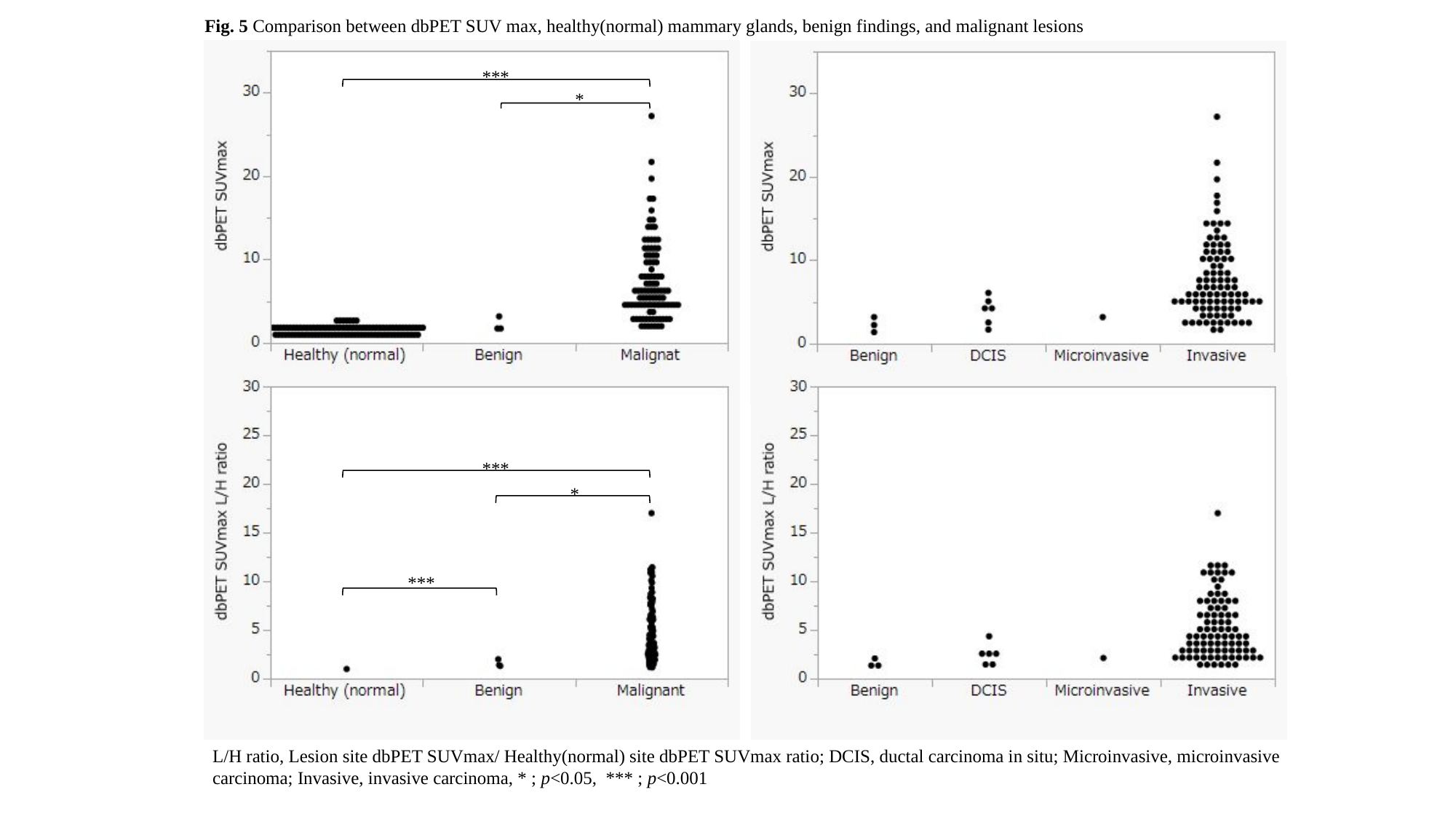

Fig. 5 Comparison between dbPET SUV max, healthy(normal) mammary glands, benign findings, and malignant lesions
***
*
***
*
***
L/H ratio, Lesion site dbPET SUVmax/ Healthy(normal) site dbPET SUVmax ratio; DCIS, ductal carcinoma in situ; Microinvasive, microinvasive carcinoma; Invasive, invasive carcinoma, * ; p<0.05, *** ; p<0.001

## Slide 6
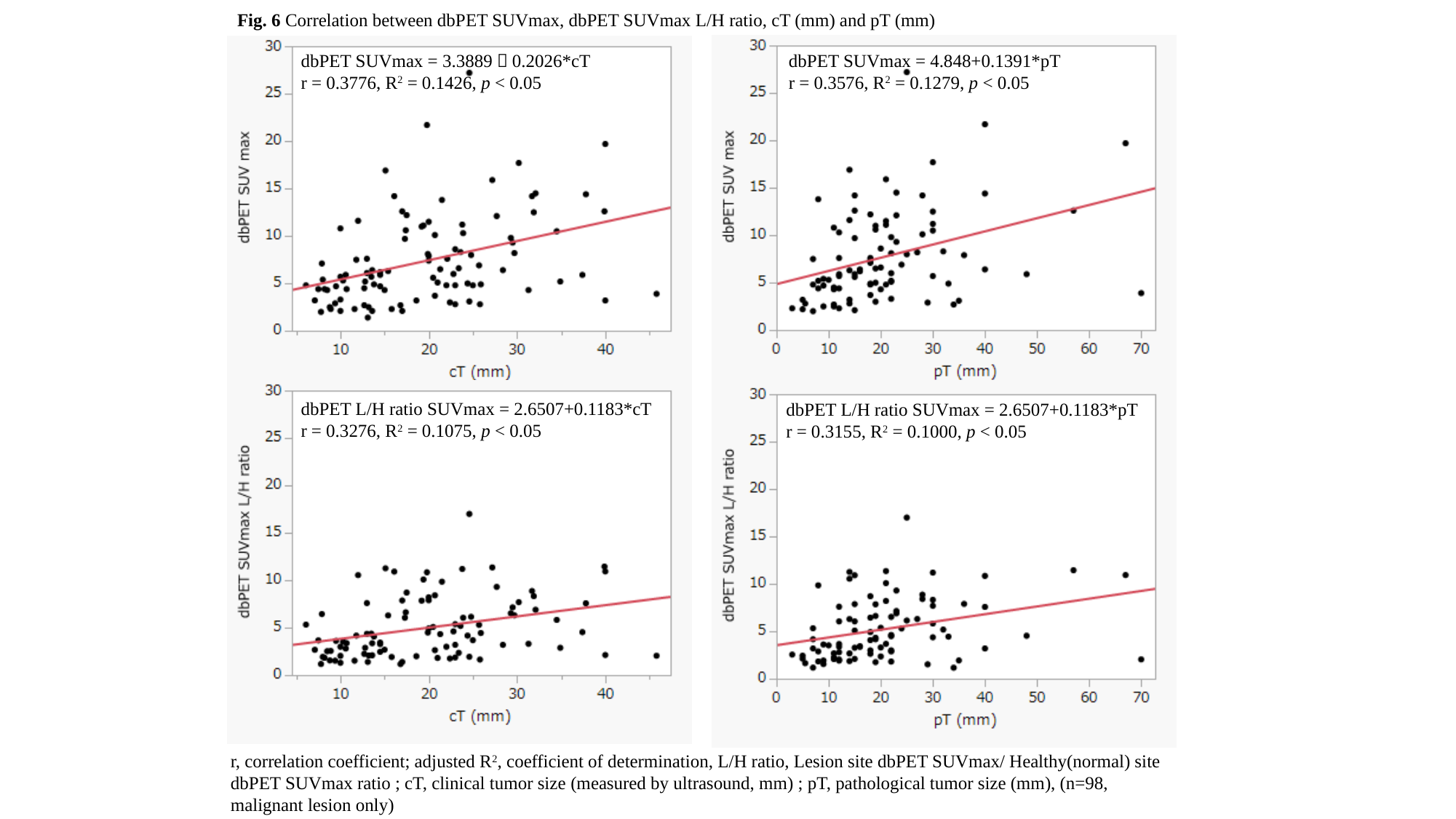

Fig. 6 Correlation between dbPET SUVmax, dbPET SUVmax L/H ratio, cT (mm) and pT (mm)
dbPET SUVmax = 3.3889＋0.2026*cT
r = 0.3776, R2 = 0.1426, p < 0.05
dbPET SUVmax = 4.848+0.1391*pT
r = 0.3576, R2 = 0.1279, p < 0.05
dbPET L/H ratio SUVmax = 2.6507+0.1183*cT
r = 0.3276, R2 = 0.1075, p < 0.05
dbPET L/H ratio SUVmax = 2.6507+0.1183*pT
r = 0.3155, R2 = 0.1000, p < 0.05
r, correlation coefficient; adjusted R2, coefficient of determination, L/H ratio, Lesion site dbPET SUVmax/ Healthy(normal) site dbPET SUVmax ratio ; cT, clinical tumor size (measured by ultrasound, mm) ; pT, pathological tumor size (mm), (n=98, malignant lesion only)

## Slide 7
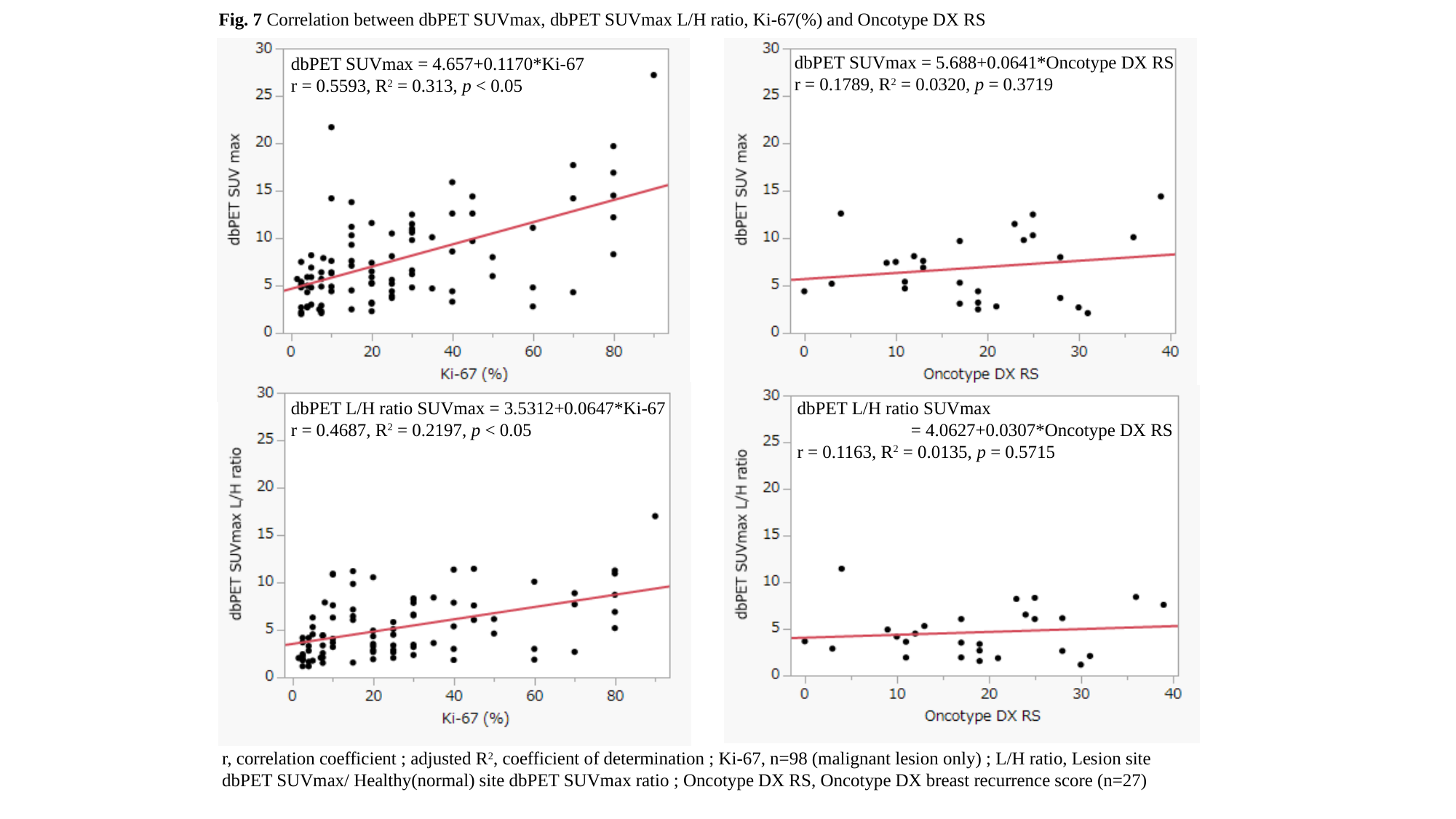

Fig. 7 Correlation between dbPET SUVmax, dbPET SUVmax L/H ratio, Ki-67(%) and Oncotype DX RS
dbPET SUVmax = 5.688+0.0641*Oncotype DX RS
r = 0.1789, R2 = 0.0320, p = 0.3719
dbPET SUVmax = 4.657+0.1170*Ki-67
r = 0.5593, R2 = 0.313, p < 0.05
dbPET L/H ratio SUVmax = 3.5312+0.0647*Ki-67
r = 0.4687, R2 = 0.2197, p < 0.05
dbPET L/H ratio SUVmax
 = 4.0627+0.0307*Oncotype DX RS
r = 0.1163, R2 = 0.0135, p = 0.5715
r, correlation coefficient ; adjusted R2, coefficient of determination ; Ki-67, n=98 (malignant lesion only) ; L/H ratio, Lesion site dbPET SUVmax/ Healthy(normal) site dbPET SUVmax ratio ; Oncotype DX RS, Oncotype DX breast recurrence score (n=27)

## Slide 8
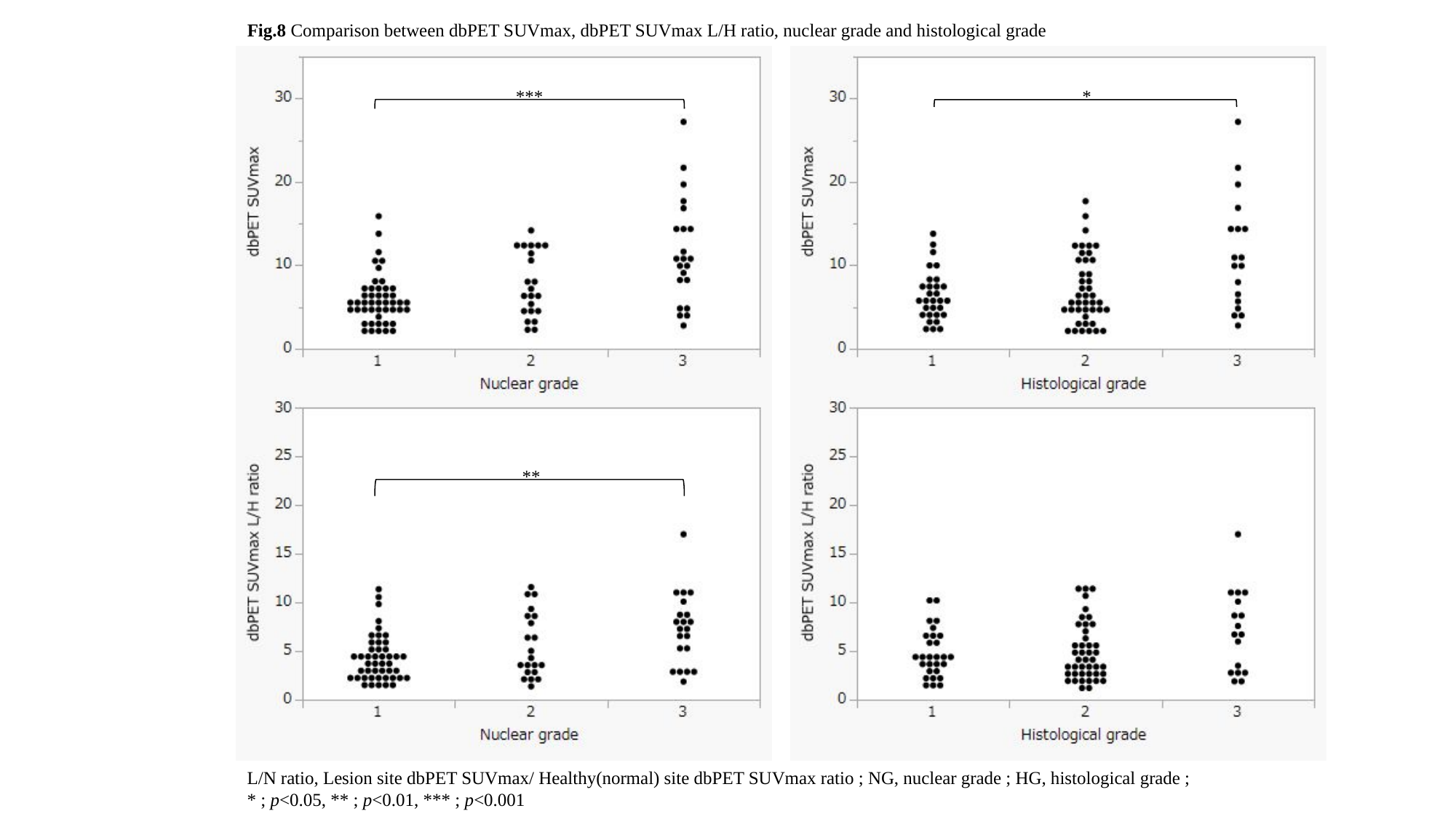

Fig.8 Comparison between dbPET SUVmax, dbPET SUVmax L/H ratio, nuclear grade and histological grade
***
*
**
L/N ratio, Lesion site dbPET SUVmax/ Healthy(normal) site dbPET SUVmax ratio ; NG, nuclear grade ; HG, histological grade ;
* ; p<0.05, ** ; p<0.01, *** ; p<0.001

## Slide 9
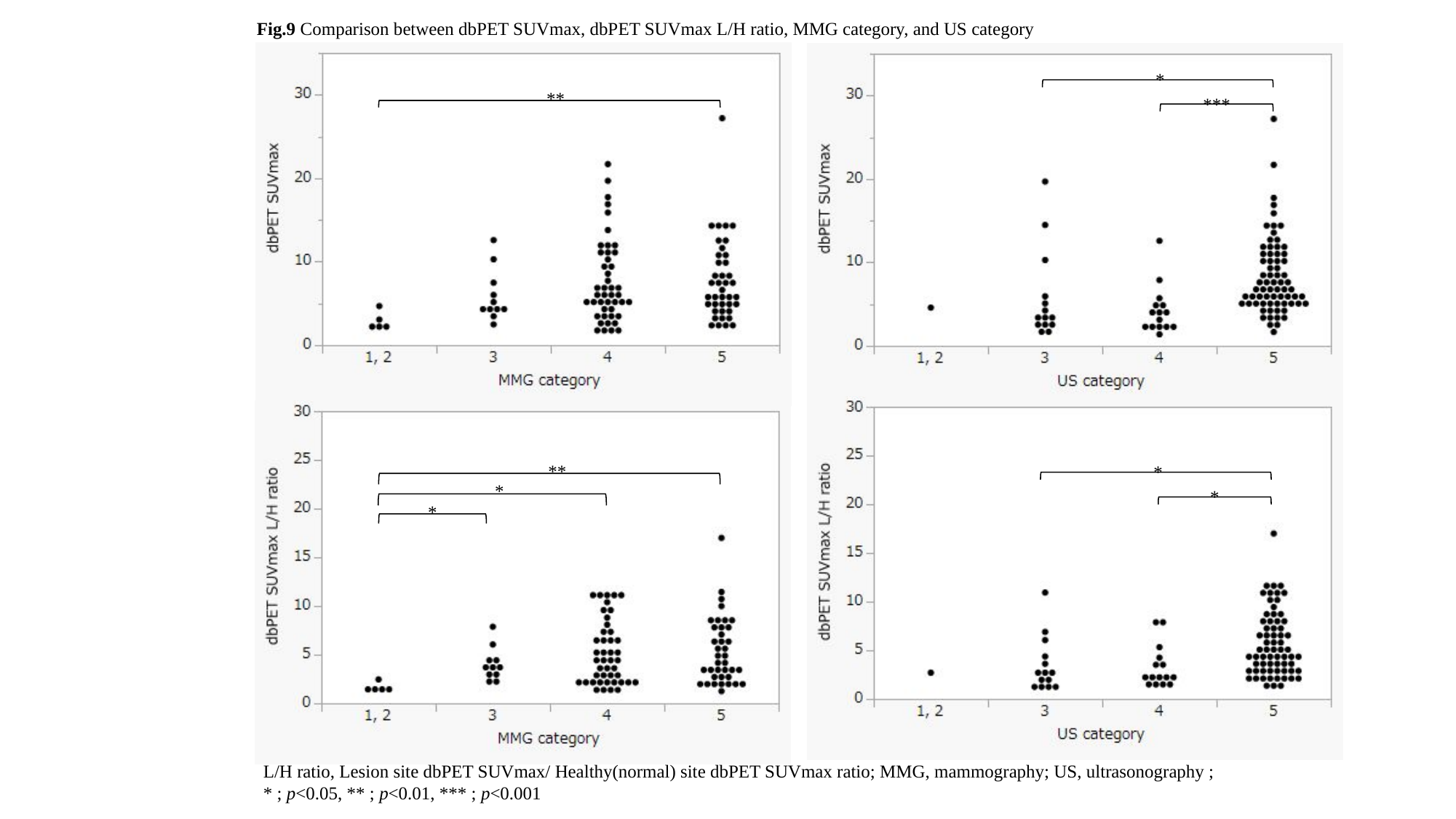

Fig.9 Comparison between dbPET SUVmax, dbPET SUVmax L/H ratio, MMG category, and US category
*
**
***
**
*
*
*
*
L/H ratio, Lesion site dbPET SUVmax/ Healthy(normal) site dbPET SUVmax ratio; MMG, mammography; US, ultrasonography ; * ; p<0.05, ** ; p<0.01, *** ; p<0.001
